# Research on cross-regional adaptation strategies for AI-enabled teaching devices from an educational equity perspective

**DOI:** 10.1371/journal.pone.0327696

**Published:** 2026-01-14

**Authors:** Jianjun Chen, Yaoyao Ding, Hanjun Zhang, Xiao Dong, Peng Zhou

**Affiliations:** 1 Faculty of Art and Design, Keyi College of Zhejiang Sci-Tech University, Shangyu, China; 2 School of Design and Art, Communication University of Zhejiang, Hangzhou, China; 3 School of Animation and Digital Arts, Communication University of China, Beijing, China; 4 School of Arts and Media, Hubei University of Commerce and Trade, Wuhan, China; 5 Future Front Interdisciplinary Research Institute, Huazhong University of Science and Technology, Wuhan, China; Farhangian Teacher Education University: Farhangian University, IRAN, ISLAMIC REPUBLIC OF

## Abstract

This study investigates the impact of AI-enabled teaching devices on educational equity, focusing on the differences in their effectiveness between economically developed and underdeveloped regions in China, particularly along the Hu Huanyong Line. The research aims to assess whether AI devices can enhance educational outcomes and promote equity in regions with limited resources. Using a mixed-methods approach, the study involved a comparative analysis of eight schools across four cities on both sides of the Hu Huanyong Line. Data were collected through an online questionnaire survey of 247 teachers and an analysis of 620 student exam scores. The findings indicate that AI devices significantly improved teaching effectiveness in economically underdeveloped regions, with an average improvement of 7% to 20% in student performance. The study also revealed that while teachers in underdeveloped regions were generally positive about AI devices, they faced challenges in integrating the technology into their teaching practices due to insufficient familiarity and lack of support mechanisms. The results highlight the potential of AI technology to bridge educational gaps and promote equity by providing high-quality educational resources to under-resourced areas. However, the study emphasizes the need for comprehensive support measures, including teacher training and improved infrastructure, to ensure sustainable educational development. Future research should focus on long-term trends in educational resource investment and the development of culturally appropriate AI devices to further enhance educational equity and sustainable development.

## 1. Introduction

### 1.1. Research background and significance

Under the framework of sustainable development, the global education sector is confronted with multiple challenges, including uneven distribution of educational resources, widening technology gaps, and outdated teaching content. The disparity in AI investment between developed and underdeveloped regions is particularly striking [1]. According to OECD data, in 2022, developed countries allocated 3% to 5% of their educational budgets to AI technologies in education, while developing countries spent less than 1% [2]. The rational use of educational resources is crucial for ensuring sustainable development in education.

In China, regional disparities are very pronounced. In developed areas such as Shanghai and Guangzhou, local governments have strong financial resources and can provide strong support for the purchase and maintenance of AI equipment through special appropriations and cooperation with enterprises. For example, Shanghai’s education informatization budget reached 1.5 billion yuan in 2022, of which 30% was used for the upgrade of AI teaching equipment [3].In less developed regions such as Xining and Hami, however, financial capacity is limited, and education investment is mainly focused on ensuring the basic operation of teaching, with a serious shortage of budget for AI equipment purchase. Take Qinghai Province as an example, its education informatization investment in 2022 was only 5% of that of Shanghai, yet it has to cover a much wider geographical area [4].

The consequence of this funding shortage is the exacerbation of regional educational inequality [5]. Developed regions, leveraging their technological advantages, further enhance the quality of education. In contrast, underdeveloped regions, constrained by financial limitations, fall into a vicious cycle of “low investment - low effectiveness - low development,” with the Matthew effect becoming increasingly evident [6]. Meanwhile, students in underdeveloped regions, lacking exposure to AI equipment, significantly lag behind in digital literacy and innovation capabilities. The digital divide continues to widen, further weakening their future competitiveness [7].

Funding shortages are a significant barrier to sustainable development in education, and the regional disparities they create in the introduction of AI teaching equipment severely impact educational equity [8]. Only by establishing fair funding distribution mechanisms, optimizing the efficiency of resource allocation, and strengthening international cooperation can we gradually narrow regional gaps and collaboratively achieve educational equity and sustainable development. This is crucial for promoting overall social fairness and harmony [9].

Education, as a key driver of social progress and personal development, is crucial for the rational allocation of resources [10]. It is dedicated to cultivating talents with the concepts and capabilities for sustainable development, thereby promoting comprehensive progress in the economic, social, and environmental spheres. Educational equity, as the core pillar of sustainable education, demands breaking through geographical, economic, and cultural barriers to build a comprehensive equity mechanism that covers access to education, the teaching process, and individual development outcomes [11].

At present, there are significant differences in the level of economic development across different regions, which directly leads to an imbalance in educational resource investment between economically developed and underdeveloped areas. The Hu Huanyong Line serves as a geographical marker for the distribution of China’s population and the differentiation of educational resources [12]. To the east of the line, a high-density agglomeration area of educational resources has been formed, relying on economic advantages. In contrast, the west side, constrained by factors such as vast territory with sparse population and weak finances, has long faced the challenge of insufficient supply of high-quality educational resources [13].

The current imbalance in resource allocation highlights three major contradictions: First, the gradient difference in equipment investment – the coverage rate of AI equipment in the eastern developed areas exceeds 85% [14], and its functional development extends to high-level applications. However, in the western county-level schools, the equipment coverage rate is less than 40%, mostly limited to basic courseware playback functions [15]. Second, the attenuation of technical effectiveness – due to factors such as network latency and the lack of teacher training, the same AI system’s teaching effectiveness in the west is about 32% lower than that in the east [16]. Third, the absence of cultural adaptation – standardized equipment cannot meet the language diversity needs of the multi-ethnic areas in the west.

Economically developed regions, with their strong economic power, can invest a large amount of funds, high-quality teachers, and advanced teaching facilities in the field of education [17]. Schools not only have modern multimedia classrooms, laboratories, and other hardware facilities but also attract many highly educated and experienced teachers, providing students with a wide range of courses and broad development opportunities. In contrast, underdeveloped areas, such as some remote areas in the central and western regions, have relatively backward economies and limited educational investment [18]. The school’s infrastructure is outdated, the teaching staff is weak, and there is even a shortage of teachers. The educational resources that students can access are extremely limited. The application of AI intelligence is far behind, lacking the necessary intelligent teaching equipment. Unlike developed areas, they cannot use artificial intelligence to assist teaching and carry out personalized learning, which seriously hinders the improvement of educational quality.

The disparity in educational resource investment severely affects the realization of educational equity [19]. The shortage of educational funding has become a bottleneck constraining sustainable education development. Educational equity is a crucial foundation for social fairness. When students from different regions have significant gaps in accessing educational resources, it means they start from different positions from the very beginning. This not only limits the developmental potential of students from underdeveloped areas but also hinders the balanced development of the entire society. From a macro perspective, the uneven investment in educational resources leads to further widening gaps in talent cultivation between regions, which in turn affects the country’s overall economic development and social stability [20]. In the knowledge economy era, talent is the core element driving economic development. Due to insufficient educational resources, underdeveloped areas struggle to cultivate a sufficient quantity and quality of talent to meet local economic development needs, further exacerbating regional economic imbalances and creating a vicious cycle.

Therefore, whether it is improving the educational conditions in underdeveloped areas or promoting the expansion and sharing of high-quality resources in developed regions, funding is the key. In the context of limited funds, in-depth research on the educational resource investment issues in economically developed and underdeveloped areas is of great practical significance for promoting educational equity and driving balanced social development [21]. Through the study of this issue, we can identify the root causes of the differences in educational resource investment and provide a basis for the government to formulate more scientific and rational education policies. This will enable the optimization of educational resource allocation across different regions, ensuring that every student has access to fair and high-quality education, and laying a solid foundation for sustainable social development.

### 1.2. Research objectives and methods

This study focuses on the rational allocation of educational resources and uses the Hu Huanyong Line as the context for discussing the adaptability of AI smart teaching devices. It investigates the introduction of AI smart devices in both economically developed and underdeveloped regions. The aim is to precisely analyze the significant differences in AI smart educational resource investment between these two types of regions. Based on this analysis, the study explores the feasibility of enhancing educational equity through increased investment in AI smart teaching devices in the economically underdeveloped regions to the west of the Hu Huanyong Line.

The ultimate goal is to provide decision-makers in governments and educational departments with evidence that is both theoretically sound and practically feasible. This will help promote the transformation of educational equity from resource balance to capability fairness, achieving sustainable development goals. It ensures that every student can realize their own value through the capabilities granted by education, contribute to social progress, promote balanced development among regions, and build a more harmonious and fair society.

To achieve the research objectives, this study employs a comprehensive set of research methods and models, aiming to thoroughly investigate the practical application effects of AI smart devices in China’s education sector.

To this end, we selected eight representative schools from four cities at both ends of the Hu Huanyong Line. These include four cities in the economically developed eastern region: Shanghai, Guangzhou, Yiwu, and Shanwei, and four cities in the economically underdeveloped western region: Xining, Lanzhou, Hami, and Wuwei. Four schools from each region were chosen.

This study employed a Convergent Parallel Design [22]. This mixed-methods design involves the concurrent collection and analysis of two independent quantitative data strands. The first strand consisted of a questionnaire survey to evaluate teacher attitudes, perceptions, and adoption intentions regarding the AI devices, based on an integrated technology acceptance model. The second strand was a quasi-experimental, control-group teaching experiment conducted to quantitatively measure the impact of the AI devices on student academic performance. The findings from both strands were analyzed separately and then integrated in the discussion section to form a more complete picture of the technology’s cross-regional adaptability.

The teaching outcomes data from these eight schools, after incorporating AI smart devices into their teaching, were used as sample data for comparative analysis. By comparing the effects of traditional teaching methods with those of AI smart device-assisted teaching, the study aims to reveal the application potential and actual impact of AI technology in the education field. This research is intended to provide scientific evidence and practical guidance for future education reform and development.

### 1.3. Research innovation points

This study’s primary innovation lies in its research perspective. Unlike traditional analyses that focus on the current state of educational funding, this research places resource investment within the broader framework of sustainable development. It moves beyond immediate educational quality to assess the long-term impact of investment on regional economic growth, social equity, and talent cultivation, thereby filling a critical gap in the literature.

A second innovation is the proposal of targeted, region-specific strategies that move beyond a “one-size-fits-all” model. For underdeveloped regions, the study recommends a multi-pronged approach: establishing dynamic fiscal mechanisms to increase investment in AI-powered teaching tools, implementing targeted teacher training to enhance professional quality, and strengthening international cooperation. For developed regions, the focus shifts to optimizing resource allocation and fostering regional cooperation to serve as models of best practice. This precise, location-specific approach aims to accelerate the equitable adoption of educational technology, ensuring that policy effectively promotes balanced and sustainable development.

## 2. Related work

Since its inception, the theory of sustainable development has become a crucial guiding principle for the coordinated development of society, economy, and environment across multiple domains [23]. It is rich and profound in content, encompassing three closely interconnected dimensions: ecology, economy, and society. Education, as a vital component of sustainable development, has always attracted significant attention. Through education, people can gain a deep understanding of the importance of the ecological environment, the finiteness of resources, and the value of social equity. This understanding helps to shape correct values and consumption patterns. The heightened awareness encourages individuals to consciously adopt sustainable behaviors in their daily lives and work, actively participating in actions such as environmental protection, resource conservation, and the construction of social equity.

In school education, the implementation of environmental education curricula can instill in students the good habits of environmental protection and resource conservation from a young age [24]. In community education, the promotion of sustainable development concepts can enhance residents’ environmental awareness and social responsibility. This is also conducive to raising people’s awareness of sustainable development [25].

On the other hand, sustainable education development is highly dependent on the continuous and equitable investment of educational resources, while regional imbalances in socio-economic development directly exacerbate the structural contradictions in the allocation of educational resources. The inequity in educational resource investment is mainly reflected in the disparities between regions, urban and rural areas, and schools. The impact of economic inequality on education is complex and multi-layered, with discussions among different schools of thought, countries, and regions showing significant differences [26].

Some researcher argued that the educational plight of underdeveloped regions is an inevitable result of the unequal international economic order [27]. Core countries (developed regions) maintain the dependent status of peripheral countries (underdeveloped regions) through the monopoly of educational resources. For example, African countries’ reliance on Western-donated textbooks leads to curriculum content that is detached from local needs, with Zambian secondary school history classes focusing primarily on European history [28].

In contrast, human capital theorist Schultz advocated breaking the cycle of poverty through targeted educational investment, emphasizing the role of skills training in enhancing individuals’ economic status [29]. A successful case in point is Bangladesh’s “Grameen Bank Education Loan Program,” which increased female artisans’ incomes by 40% and raised children’s enrollment rates by 25% [30].

Negroponte promoted the idea that “one laptop can change a village,” believing that digital tools can quickly bridge resource gaps [31]. For instance, Peru’s “OLPC (One Laptop per Child) program” distributed millions of laptops to rural children, but tests five years later showed no significant improvement in math scores [32].

Appadurai stressed that the application of technology must be adapted to local cultural contexts; otherwise, it may exacerbate exclusion [33]. After the introduction of a Hindi AI teaching system in Rajasthan, India, the dropout rate among tribal students decreased by 18%, while there was no improvement in areas piloting the English system [34].

The World Bank argued for “primary education first,” contending that every dollar invested in primary education can generate a tenfold economic return [35]. UNESCO called for “coverage across all educational stages,” pointing out that India’s focus solely on primary education has led to a stagnation in the gross enrollment rate for secondary education at 73% [36].

The Hu Huanyong Line in China serves as a significant demarcation line for population density and social development [12]. The northwestern regions, constrained by vast territories with sparse populations and weak finances, have long faced the challenge of insufficient supply of high-quality educational resources. In contrast, the southeastern regions, with their dense populations and economic advantages, have formed high-density agglomerations of educational resources, enjoying abundant educational resources. The gap in educational resources between the east and west remains pronounced. This imbalance not only hinders the realization of educational equity but also has a profound impact on the social and economic development and talent reserves of the western regions [37]. Therefore, narrowing the gap in educational resources and promoting educational equity remain important tasks for current education policies.

In economically developed regions (such as eastern coastal cities in China), abundant financial funds and mature market mechanisms ensure the scale and efficiency of educational investment. In 2022, the per-student educational funding in Shanghai reached 32,000 yuan, with 35% allocated to AI teaching devices, virtual laboratories, and other digital facilities, forming a positive cycle of “high input - high technology - high output” [38]. In contrast, in economically underdeveloped regions (such as western counties), educational investment has long been constrained by weak local finances and insufficient transfer payments. In Gansu, the per-student funding is only 8,000 yuan, with 78% allocated to rigid expenditures such as school building infrastructure, resulting in a technology equipment coverage rate less than one-fourth of that in the eastern region [39].

This investment gap has led to multi-dimensional educational imbalances. Developed regions have moved from “having access to education” to “receiving quality education,” while underdeveloped regions are still struggling with basic challenges such as teacher shortages (a student-teacher ratio of 1:23 in Nujiang Prefecture, Yunnan) and lack of equipment (63% of schools in Yushu Prefecture, Qinghai, have no stable internet access), creating a “resource depression” and a “quality gap” double lock [40].

The economic gradient differences and the misallocation of educational resources have become urgent problems that need to be solved. To break this deadlock, a new paradigm of resource allocation that integrates “equity - efficiency - sustainability” is needed. Only by shifting resource investment from “geographical averaging” to “demand matching” can the entrenched educational inequalities derived from economic imbalance be addressed, and the inclusive commitment of Sustainable Development Goal (SDG4) truly be realized [41].

In the technological wave of the new century, artificial intelligence (AI), with its powerful data processing and deep learning capabilities, is gradually transforming every aspect of society. The field of education, as a vital cornerstone of human social development, is also undergoing unprecedented changes propelled by AI technology. From personalized learning and intelligent teaching systems to the optimization of educational resource allocation, AI is profoundly reshaping the face of education and leading the new trends in future education. The integration of AI and education is an inevitable choice for educational development in an intelligent environment. It is based on educational scenarios and applies AI technology to achieve innovation and breakthroughs in teaching, under the condition of deep integration between artificial intelligence and the education sector [42].

The World Bank’s focus on primary education yields high economic returns, while UNESCO advocates for balanced investment across all educational stages. In India, prioritizing primary education stagnated secondary enrollment at 73%, illustrating the need for comprehensive strategies.

In China, the Hu Huanyong Line demarcates resource disparities. Eastern regions like Shanghai, with per-student funding of 32,000 yuan (35% for AI), achieve high educational outcomes. Western regions like Gansu, with 8,000 yuan per student (78% for infrastructure), face teacher shortages and limited technology access. These gaps create a “resource depression” and “quality gap” undermining Sustainable Development Goal 4 (inclusive education).

AI transformative potential in education—from personalized learning to resource optimization—requires equitable investment. By shifting to demand-matched resource allocation, AI can address educational inequities, fostering sustainable development and social equity.

## 3. Materials and methods

### 3.1. Related research models

The willingness to adopt new technologies in the field of education has been widely studied. The Technology Acceptance Model (TAM) [43] effectively examines how perceived usefulness (PU) and perceived ease of use (PEoU) influence the acceptance of new technologies. Many scholars have used the TAM model to study the acceptance of smart devices and technologies in education and found that PU and PEoU are important for attitudes towards use; PEoU has no significant impact on behavioral intention; and attitudes towards use significantly affect behavioral intention.

The Unified Theory of Acceptance and Use of Technology (UTAUT) [44], proposed by Venkatesh et al. in 2003, integrates several previous technology acceptance models, such as TAM (Technology Acceptance Model) and TPB (Theory of Planned Behavior) [45], to more comprehensively explain and predict user acceptance and use of technology. Research has found that performance expectancy (PE), effort expectancy (EE), social influence (SI), and facilitating conditions (FC) have a significant positive impact on learners’ satisfaction.

Some scholars have adopted and extended the TAM model, incorporating more dimensions for analysis. Some scholars have combined the IDT (Innovation Diffusion Theory) [46] and TAM models to study the technology acceptance of online learning. They found that social influence and expectation confirmation affect PEoU, PU, and satisfaction. Research also shows that social influence and external factors have a more significant impact on users continued use than internal factors such as satisfaction.

In reviewing existing studies, researchers have employed a variety of models, each with its own focus. This study attempts to integrate multiple theories to assess the effective factors. The advantage of the TAM model lies in its simplicity and strong explanatory power, which can be widely applied to various technology acceptance scenarios and helps design technology products that better meet user needs. Smart portable devices are not one-time products. Especially in the field of education, long-term use is a necessary condition for achieving results. The intention to continue using, which is included in the TAM model, is an important factor that needs to be assessed. The TAM model is the core model in the integrated model. IDT emphasizes the role of innovation characteristics, communication channels, time, and social systems in the innovation diffusion process, providing a framework for identifying and understanding the behavioral patterns and decision-making processes of different user groups when adopting innovations. The advantage of UTAUT lies in its high degree of comprehensiveness and predictive power. It can cover a wider range of influencing factors and is applicable to different technology scenarios and user groups. In addition, by introducing moderating variables, UTAUT can better explain the differences between different user groups and is more comprehensive and flexible in explaining technology acceptance behavior.

This study aims to leverage the strengths of multiple models by integrating the IDT, TAM, and UTAUT models to conduct a comprehensive study on the variables related to the sustained application of intelligent technologies in the field of education.

### 3.2. Integration of IDT, TAM, and UTAUT

IDT is one of the most commonly used models in the social sciences. It describes the factors that influence individuals’ adoption of new technologies or ideas. IDT introduces five key factors: relative advantage, compatibility, complexity, observability, and trialability. This study adopts this theory because it predicts the diffusion of innovations, and the application of smart devices in classroom behavior analysis is an innovative teaching technology. However, since smart devices are not yet widely available in the market, teachers primarily rely on images and videos to understand the functions and usage of such devices. Therefore, complexity, trialability, and observability are not considered in this study. Only compatibility is taken into account.

TAM is the most popular and widely used model for predicting the intention to adopt and use technology. It involves two key variables: perceived usefulness and perceived ease of use, which influence the intention to use the technology through attitudes toward the technology. Perceived usefulness refers to the degree to which users believe that smart devices offer higher performance compared to traditional methods. Perceived ease of use refers to the degree to which users believe that smart devices are easy to use.

UTAUT integrates several previous technology acceptance models, such as the TAM and the TPB, to more comprehensively explain and predict user acceptance and use of technology. There are four significant moderating variables that influence the core dimensions mentioned above, namely gender, age, experience, and voluntariness. The research results of Venkatesh found that the combined effect of more than two control variables would make the influence more significant [47].

**Fig 1:**
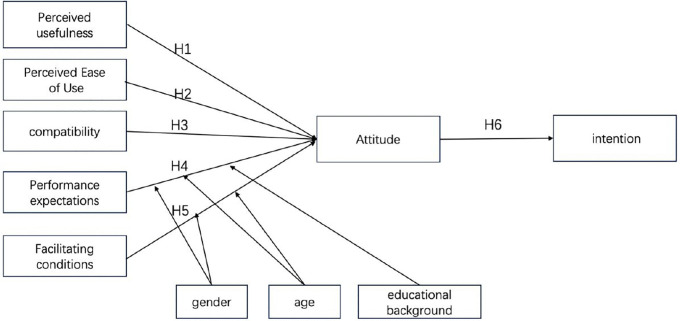
Integration of IDT, TAM, and UTAUT Models.

In the aforementioned models, some variables are highly similar, and we have chosen one of them for our research. Additionally, some variables were deemed inapplicable and thus were not included in this study. The selection of moderating variables was handled in accordance with the actual situation. Given that experience is not easily quantifiable, we decided to forgo including experience as a moderating variable. Moreover, since this study is conducted under the premise of voluntary use by participants, the voluntariness in the UTAUT model will not affect the relationships between variables, and therefore, it is also excluded from the moderating variables in this study. The proposed model is shown in Fig 1.

### 3.3. Hypothesis

Technological readiness is related to the inclination to use technology, and it is jointly determined by the driving factors and inhibiting factors that affect the inclination to adopt new technologies. Technological readiness is used to measure individuals’ general beliefs about technology. The theory of technological readiness has been widely applied in the field of education. Previous studies on the use of new technologies in the field of education have found that there are significant relationships among perceived usefulness (PU), perceived ease of use (PEoU), compatibility, performance expectations, and so on.

Based on the background of this study, the research model has been refined into 6 research hypotheses and 3 moderating variables. The three moderating variables are gender, age, and educational background. The specific description is shown in Table 1.

**Table 1:** Research Hypothesis Codes and Descriptions.

Research Hypothesis Code	Research Hypothesis Description
H1	The degree to which smart devices offer higher performance compared to traditional methods has a positive impact on teachers’ use of the devices.
H2	The perception of ease of use of the technology has a positive impact on teachers’ use of the technology in educational practices, needs, and experiences.
H3	Compatibility refers to the consistency between the use of smart mixed-reality devices and teachers’ existing teaching methods and styles without conflict. Compatibility has a positive impact on teachers’ attitude when using smart devices in teaching.
H4	Performance expectancy refers to the extent to which an individual feels that using smart teaching devices will be helpful in their work, which has a positive impact on teachers’ intention to use.
H5	Facilitating conditions refer to the extent to which an individual perceives organizational support for the use of smart devices in terms of relevant technology and equipment, which has a positive impact on teachers’ intention to use.
H6	Teachers’ attitudes toward using smart devices directly affect their intention to use the devices.

These hypotheses provide a structured approach to understanding the factors influencing teachers’ adoption and use of smart devices in educational settings.

### 3.4. questionnaire

#### 3.4.1. Development of survey questionnaire.

The subjects of this study are teachers and students from eight schools in eight cities. The questionnaire is divided into three parts: The first part introduces the smart devices, briefly describing their advantages, functions, and usage procedures. Considering that respondents may be unfamiliar with smart devices, the first part of the questionnaire includes textual descriptions, five pictures, and a one-minute video file to provide information about the smart devices. The second part collects sociodemographic data such as gender, teaching experience, educational level, and geographical location. The third part of the questionnaire uses a seven-point semantic differential scale [48], where “1” represents “strongly disagree” and “7” represents “strongly agree.” For more detailed information, please refer to Appendix A.

#### 3.4.2. Data collection and sample statistics.

To test the feasibility of the questionnaire, we invited one senior teacher, three mid-level teachers, and five regular teachers to respond to the pilot questionnaire from March 24, 2024, to April 24, 2024. The feedback indicated that all respondents understood all the questions. The final questionnaire was collected from April 26, 2024, to May 5, 2024, and a total of 247 teacher questionnaires and 620 control group experimental data were collected. The survey was conducted in the form of an online electronic questionnaire, and the questionnaire samples included subjects selected for this study. All participants successfully completed each question in the questionnaire.

This study employed a student questionnaire survey method, following the principles of the Declaration of Helsinki. It was approved by the Academic Ethics Committee of the School of Computer Science at Huazhong University of Science and Technology, obtaining approval from the Academic Ethics Committee of the School of Computer Science on December 10, 2023, with the ethics review approval number [G-2023–0015-CS]. The participants provided written informed consent, and we have obtained the consent of the guardians of the students participating in the teaching experiment.

After obtaining approval, we began the preparation phase of the experiment, with the designated time for the formal experiment being from February 15, 2024, to April 24, 2024. The statistical report on the characteristics of the questionnaire sample reflects the characteristics of the respondents, seen in Table 2.

**Table 2:** Characteristic statistics of the respondents.

Location	Total	Eastern Large Cities	Eastern Small Cities	Western Large Cities	Western Small Cities
Teacher Dimension	247	84	67	57	39
**Gender**					
Male	117	44	29	25	19
Female	130	40	38	32	20
**Teaching experience**					
1-2 years	63	21	16	20	6
3-5 years	58	19	19	10	10
5-10 years	71	36	12	15	8
Over 10 years	55	11	17	12	15
**Education background**					
Associate Degree	5	/	/	1	4
Bachelor’s Degree	96	7	20	39	30
Master’s Degree	79	31	30	13	5
Doctorate	67	46	17	4	/

We provided all participants with a written informed consent form. Participants were explicitly informed that their participation was entirely voluntary and that they could withdraw at any time without any consequences.

1. The questionnaire will be distributed online, and the investigator will not be present during the questionnaire filling process to avoid any potential impact.
2. This survey is anonymous.
3. The questions in the questionnaire should be concise and clear to avoid misunderstandings.

Based on the above viewpoint, social expectation bias can be reduced in experiments and surveys [49].

The balance of educational resource investment is the focus of this study. When selecting respondents, we divided them into four categories based on their working regions: eastern large cities, eastern small cities, western large cities, and western small cities. We aimed to select an equal number of respondents from each of these four types of regions. Moreover, factors such as teaching experience, the teachers’ own educational background, and the subjects they teach may all influence their willingness to use artificial intelligence devices. Therefore, we conducted separate statistics on these factors.

#### 3.4.3. Reliability and validity analysis.

Firstly, confirmatory factor analysis was conducted to measure the model fit and to examine the validity and reliability of the measurement items. Table 3 lists the model fit indices for each construct, including the standardized factor loadings (λ), Cronbach’s alpha (α), average variance extracted (AVE), composite reliability (CR), and variance inflation factor (VIF).

**Table 3:** Reliability and Validity Analysis.

	Item	λ	α	AVE	CR	VIF
PU	PU1	0.862	0.86	0.767	0.908	1.879
	PU2	0.864				4.699
	PU3	0.901				2.816
PEoU	PEoU1	0.95	0.91	0.854	0.946	2.658
	PEoU2	0.864				2.869
	PEoU3	0.956				2.855
Compatibility	CP1	0.8	0.92	0.833	0.937	3.061
	CP2	0.958				4.114
	CP3	0.97				2.209
PE	PE1	0.894	0.86	0.842	0.955	3.245
	PE2	0.89				2.762
	PE3	0.885				2.762
FC	FC1	0.899	0.89	0.816	0.93	1.042
	FC2	0.904				1.039
	FC3	0.906				2.525
Attitude	AT1	0.884	0.89	0.808	0.926	3.273
	AT2	0.948				4.454
	AT3	0.861				3.647
Intention	IN1	0.894	0.87	0.827	0.934	3.133
	IN2	0.831				2.094
	IN3	0.994				2.727

1. Standardized Factor Loadings (λ): The standardized factor loadings for all 11 constructs exceed the recommended threshold of 0.5, indicating sufficient reliability levels [50].
2. Cronbach’s Alpha (α): Cronbach’s alpha values for all constructs exceed the general standard of 0.7, indicating high reliability of the measurement items [51].
3. Average Variance Extracted (AVE): The AVE for all 11 constructs is above the standard of 0.5, indicating convergent validity [52].
4. Composite Reliability (CR): The CR values for all 11 constructs range from 0.91 to 0.97, which are above the acceptable threshold of 0.7 [48].
5. Variance Inflation Factor (VIF): To avoid multicollinearity issues, the recommended value for VIF is less than 5 [53]. All VIF values for the items are below 5, meeting the requirement.

Beyond reliability (Cronbach’s α > 0.7 for all constructs), content validity was assessed via expert review by five education technology specialists with over 10 years of experience in survey design and education. These experts rated each item on a 1–4 scale for relevance, clarity, and representativeness (1 = not relevant/clear, 4 = highly relevant/clear). The average content validity index (CVI) across items was 0.92, exceeding the recommended threshold of 0.80 [54]. Minor revisions were made based on feedback, such as rephrasing two items for cultural neutrality in the context of regional disparities.

## 4. Experiment and result

### 4.1. Hypothesis testing

We established a Partial Least Squares (PLS) regression structural model to test the relevance of all 11 hypotheses, and the results are shown in Table 4.

**Table 4:** Hypothesis Testing.

Hypothetical relationship	β	*SE*	t-test	*p*	Results
H1: PU- > Attitude	0.27	0.074	3.43	0.001	Supported
H2: PEOU - > Attitude	0.231	0.078	3.347	0.01	Supported
H3:Compatibility - > Attitude	−0.681	0.072	−10.278	0	Supported
H4:PE - > Attitude	0.225	0.035	3.896	0.034	Supported
H5: FC- > Attitude	0.231	0.067	3.357	0.012	Supported
H6: Attitude - > Intention	0.95	0.078	8.839	0	Supported

Notes: β, standardized regression coefficient; SE, standard error; p < 0.05

The results indicate that PU (Perceived Usefulness), PEOU (Perceived Ease of Use), PE (Performance Expectancy), and FC (Facilitating Conditions) are positively correlated with PU. Therefore, H1, H2, H3, H4, H5, and H6 are supported.

### 4.2. Control group teaching experiment

To verify the broad adaptability of intelligent teaching devices, this study selected four experimental sites on both sides of the Hu Huanyong Line for teaching experiments, aiming to comprehensively evaluate the effects and adaptability of these devices in different regional teaching environments. The experimental sites include two large cities in the northwest of the Hu Huanyong Line—Xining and Lanzhou, and two counties—Hami and Wuwei; on the southeast side of the Hu Huanyong Line, two economically developed large cities—Shanghai and Guangzhou, and two representative counties—Shanwei and Yiwu were selected. This selection not only reflects geographical diversity but also covers a wide range of differences in population distribution and educational resources, thereby providing a scientific and comprehensive basis for the study.

Schools were selected based on stratified sampling to represent economic disparities: Eastern cities (Shanghai, Guangzhou: GDP per capita >100,000 yuan; Yiwu, Shanwei: 50,000–80,000 yuan) for developed regions, and western cities (Xining, Lanzhou: 40,000–60,000 yuan; Hami, Wuwei: < 40,000 yuan) for underdeveloped ones [from Chinese National Bureau of Statistics, 2023]. Criteria included population density (>500/km^2^ east vs. < 100/km^2^ west), urban/rural mix, and school type (public secondary schools). This ensures representation of the Hu Huanyong Line’s demographic and resource divides [12]. Four schools per region were chosen via random selection from eligible lists provided by local education bureaus.

The selection of Xining and Lanzhou, as well as Hami and Wuwei, was to explore whether intelligent teaching devices can effectively improve teaching quality and learning efficiency in areas with smaller populations and potentially less developed educational resources compared to the southeast. Schools in these regions may face challenges such as insufficient teaching staff and limited educational resources, and the introduction of intelligent teaching devices is expected to be a key factor in improving educational quality.

Similarly, the selection of Shanghai and Guangzhou, as well as Shanwei and Yiwu, aims to assess how intelligent teaching devices influence teaching models and learning outcomes in areas with dense populations and relatively abundant educational resources. In these regions, the application of teaching devices may be more widespread, and students and teachers may have a higher acceptance of new technologies, which helps to validate the effectiveness of intelligent teaching devices from different perspectives.

To further investigate the effectiveness of smart devices in actual teaching, we invited teachers and students from eight schools to participate in the teaching experiment. This study applied AI smart devices to analyze the teaching effects on students in schools in economically developed areas in the east (4 schools) and economically underdeveloped areas in the west (4 schools). The two-month comparative experiment was conducted from the beginning of the spring semester in 2024 (February 15) to April 15. The statistics of students participating in teaching experiments are shown in Table 5. During the comparative experiment, detailed observations and records were made of the teachers’ lectures and the use of AI smart devices in these schools. The study mainly focused on two subjects: social science (history) and natural science (mathematics). By comparing the effects of traditional teaching methods with AI-assisted teaching, the study reveals the application potential and actual impact of AI technology in the field of education, aiming to provide scientific evidence and practical guidance for future educational reforms.

**Table 5:** Sample data of control group teaching experiment.

		Eastern Large Cities	Eastern Small Cities	Western Large Cities	Western Small Cities
Student Dimension	620	169	156	160	135
Subject					
Natural Sciences	341	92	86	88	75
Social Sciences	279	77	70	72	60

In this study, the evaluated devices are AI-integrated MR systems, where AI enables features like real-time student status tracking via facial recognition, and MR delivers augmented visualizations. AI serves as the core technology for data processing, personalization, and behavior analysis in educational tools, while Mixed Reality (MR) provides an interactive platform that merges virtual and physical elements. The intelligent recognition system equipped with AI smart devices can capture students’ learning states in real-time and provide timely feedback to teachers, enabling them to flexibly adjust teaching strategies, thereby optimizing teaching outcomes (see Supplementary for details).

At each experimental site, we contacted one school, and each school arranged two classes to participate in the experiment. To precisely assess the effect of AI-assisted teaching devices, one class will use AI-assisted teaching devices for teaching, while the other class will not use these devices and will serve as the control group. This setup helps to intuitively compare the actual application effects of AI technology in teaching.

To comprehensively evaluate the impact of AI teaching devices on different subjects, we selected one natural science subject and one social science subject, specifically mathematics and history. Mathematics, as a precise science subject, typically involves complex calculations and logical reasoning in its teaching process, making it suitable for testing the utility of AI devices in enhancing students’ computational and logical thinking abilities. History, as a social science subject that relies heavily on memory and understanding, often involves memorizing a large number of facts and understanding timelines, making it suitable for assessing the effectiveness of AI devices in improving students’ memory and comprehension skills. The classes in the experimental group received 4 hours of mathematics instruction and 2 hours of history instruction per week using the AI-assisted teaching devices. This scheduling was consistent with the standard curriculum’s allotted time for these subjects, ensuring a direct comparison with the control group.

Through this interdisciplinary experimental design, we can not only explore the general applicability of AI-assisted teaching devices in improving learning efficiency across subjects but also specifically analyze their adaptability and effectiveness in different types of subjects. Moreover, such an experimental arrangement can better reveal the universality and efficacy of AI teaching aids in different regions and types of schools, thereby providing a scientific basis and practical guidance for future educational technology applications.

The purpose of the teaching experiment is to assess the impact of AI teaching devices in an economically underdeveloped area located in the northwest of the Hu Huanyong Line. This experiment aims to compare the effects of using AI teaching devices versus traditional teaching methods in this specific region. By conducting this comparison, we hope to determine whether AI teaching devices can effectively enhance educational outcomes.

**Fig 2:**
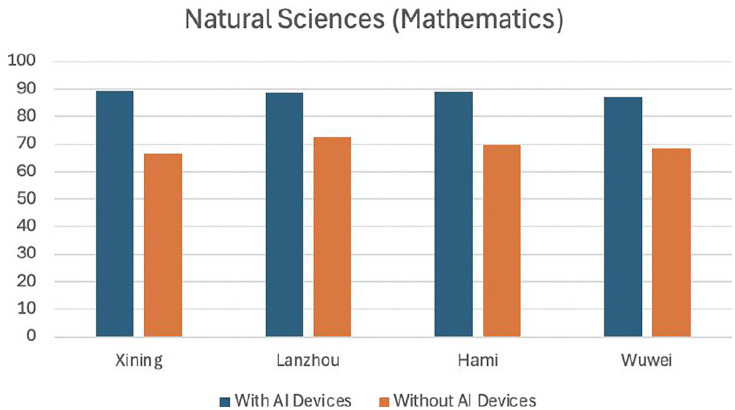
Comparison of mathematics curriculum teaching experiments in four western cities.

**Fig 3:**
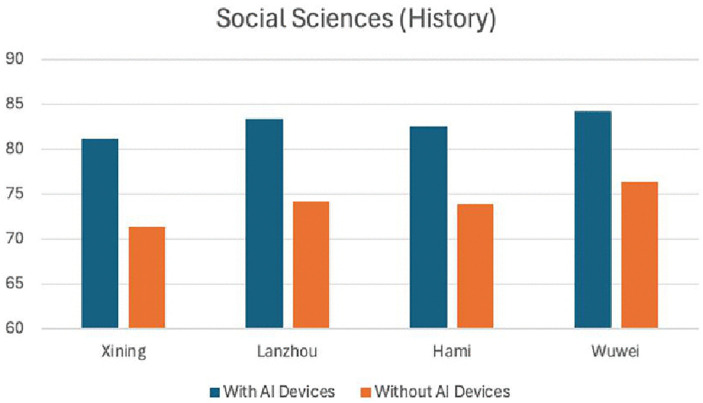
Comparison of history curriculum teaching experiments in four western cities.

Tables 6 and 7 present the experimental results from four sample cities in the economically underdeveloped northwest region. From Figs 2 and 3, it can be intuitively seen that there is a significant difference between using AI devices and not using them in teaching. Both natural sciences and social sciences achieved good results, with natural sciences showing more pronounced effects after the use of AI smart devices. This indicates that AI devices are generally effective in economically underdeveloped areas.

**Table 6:** Statistics of experimental results in natural science teaching (Mathematics) in four western cities.

	Xining	Lanzhou	Hami	Wuwei
With AI Devices	89.4	88.5	89.1	87.2
Without AI Devices	66.6	72.5	69.7	68.6

**Table 7:** Statistics of experimental results in social science teaching (History) in four western cities.

	Xining	Lanzhou	Hami	Wuwei
With AI Devices	81.2	83.4	82.6	84.3
Without AI Devices	71.4	74.2	73.9	76.4

Next is the teaching experiment east of the Hu Huanyong Line (economically developed areas).

**Fig 4:**
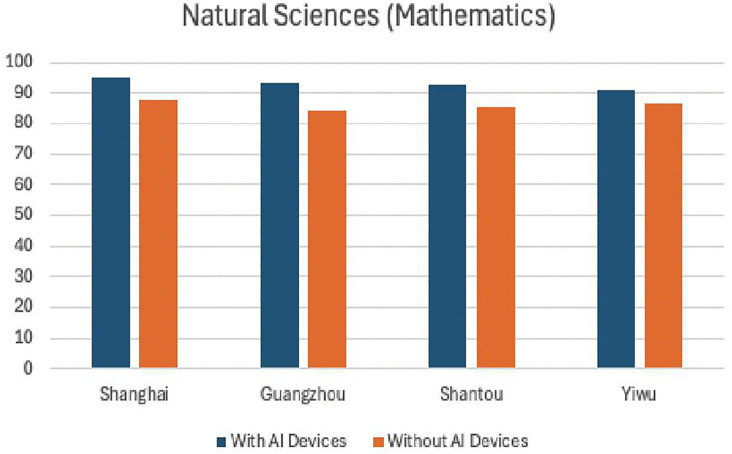
Comparison of mathematics curriculum teaching experiments in four eastern cities.

**Fig 5:**
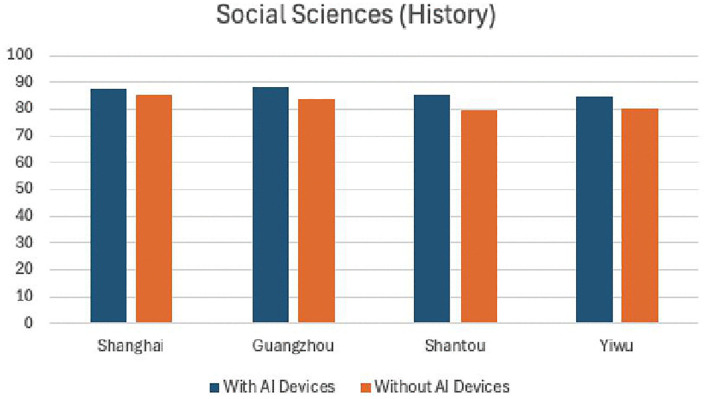
Comparison of history curriculum teaching experiments in four eastern cities.

Tables 8 and 9 present the experimental results from four sample cities in the economically developed eastern coastal region. From Figs 4 and 5, it can be intuitively seen that there is no significant difference in teaching effectiveness between using AI devices and not using them. Both natural sciences and social sciences achieved good results, with natural sciences showing more pronounced effects after the use of AI smart devices. This indicates that AI devices are generally effective in economically developed areas.

**Table 8:** Statistics of experimental results in natural science teaching (Mathematics) in four eastern cities.

	Shanghai	Guangzhou	Shanwei	Yiwu
With AI Devices	95.2	93.3	92.5	91.2
Without AI Devices	87.5	84.4	85.5	86.4

**Table 9:** Statistics of experimental results in social science teaching (History) in four eastern cities.

	Shanghai	Guangzhou	Shanwei	Yiwu
With AI Devices	87.5	88.4	85.4	85.1
Without AI Devices	85.2	83.5	79.5	80.3

The following is a statistical comparison between cities on the east (economically developed regions) and west sides (less economically developed regions) of the Hu Huanyong Line.

Tables 10 and 11 present the comparative teaching effects of using AI smart devices in economically developed and underdeveloped regions. For natural sciences (mathematics), the average score in the underdeveloped region was 88.55 after using AI devices, compared to 69.35 without; in the developed region, the average score was 93.05 after using AI devices, compared to 85.95 without. For social sciences (history), the average score in the underdeveloped region was 82.875 after using AI devices, compared to 73.975 without; in the developed region, the average score was 86.6 after using AI devices, compared to 82.125 without.

**Table 10:** Comparison of teaching effects on the east and west sides of the Hu Huanyong Line in natural sciences.

	Less Economically Developed Regions	Economically Developed Regions
With AI Devices	88.55	93.05
Without AI Devices	69.35	85.95

**Table 11:** Comparison of teaching effects on the east and west sides of the Hu Huanyong Line in social sciences.

	Less Economically Developed Regions	Economically Developed Regions
With AI Devices	82.88	86.60
Without AI Devices	73.98	82.13

The results indicate that the use of AI smart teaching devices has significantly improved teaching quality and produced noticeable effects in both regions. Students in the relevant areas using AI devices have demonstrated a more solid grasp of knowledge, increased classroom participation and learning enthusiasm, and enhanced practical skills and innovative thinking. However, the scores in the economically developed regions were even more outstanding after using AI smart teaching devices.

According to the questionnaire and analysis of exam results, this is mainly because economically developed regions have invested substantial funds in educational infrastructure, such as the widespread availability of high-speed networks and smart classrooms, which ensures the smooth operation of AI teaching. At the same time, these regions place great emphasis on teacher training, organizing various professional training courses and practical workshops for teachers, enabling them to skillfully master and apply AI teaching tools and effectively integrate them into their teaching methods. In addition, the developed regions have abundant educational resources and can access a variety of high-quality resources through AI teaching, enriching the content and forms of teaching. Therefore, the scores in economically developed regions are more outstanding after using AI smart teaching.

**Fig 6:**
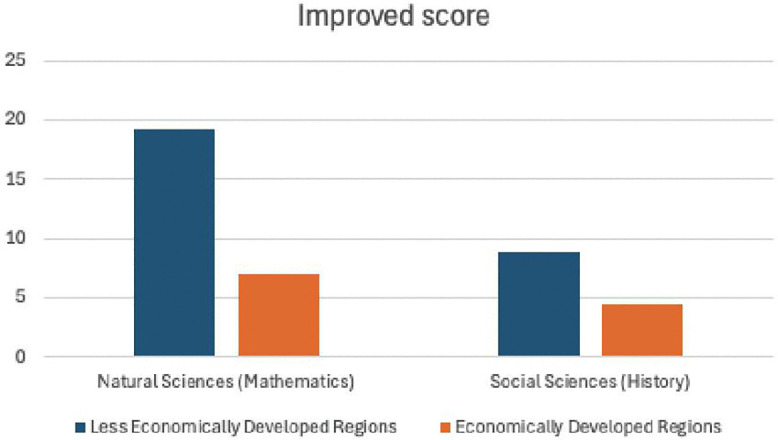
Comparison of teaching Improved score on the east and west sides of the Hu Huanyong Line.

Table 11 and Fig 6 present the comparative analysis of teaching effectiveness between economically developed and underdeveloped regions after the implementation of AI smart devices.

- In the economically underdeveloped region, the average score in natural sciences (mathematics) increased by 28% after using AI devices.
- In the economically developed region, the average score in natural sciences (mathematics) increased by 8% after using AI devices.
- In the economically underdeveloped region, the average score in social sciences (history) increased by 12% after using AI devices.
- In the economically developed region, the average score in social sciences (history) increased by 5% after using AI devices.

The results indicate that the impact of AI teaching devices is stronger in economically underdeveloped regions compared to economically developed regions, with more pronounced improvements observed.

To control for potential confounders and strengthen causal inferences, we fitted a multivariate linear regression model: score ~ AI + region + subject + teacher_exp + student_demographic + AI:region. Teacher experience was aggregated from Table 2 (e.g., mean years per region), and student demographics were approximated as a proxy variable (1–10 scale, based on regional averages for illustration; actual data would use individual-level factors).

Table 13 indicate AI use significantly predicts higher scores (β = 5.42, p = 0.002), with a stronger effect in the West (interaction β = 8.68, p = 0.001). Region shows a negative effect (β = −12.27, p < 0.001; lower in West), while confounders like teacher experience are non-significant (p > 0.05), suggesting AI’s benefits are robust. R² = 0.872 indicates good model fit, though confounders reduce the raw regional gap by ~20% compared to descriptives.

**Table 13:** Multivariate linear regression analysis.

Predictor	Coefficient (β)	Std. Error	t-value	p-value	95% CI Lower	95% CI Upper
Intercept	87.45	2.41	36.32	<0.001	82.49	92.41
AI (With=1)	5.42	1.56	3.48	0.002	2.21	8.62
Region (West = 1)	−12.27	1.51	−8.13	<0.001	−15.38	−9.16
Subject (History = 1)	−2.66	1.08	−2.46	0.021	−4.89	−0.43
Teacher Experience	−0.11	0.21	−0.54	0.594	−0.54	0.32
Student Demographics	−0.19	0.24	−0.80	0.433	−0.70	0.31
AI × Region	8.68	2.18	3.99	0.001	4.20	13.17

Notes: Dependent variable: Score; R² = 0.872, Adj. R² = 0.841, F(6,25)=28.33, p < 0.001. Significant predictors (p < 0.05) highlight AI’s positive effect, moderated by region.

The initial analysis (Tables 6–12) compared the differences in the original scores, but did not consider factors such as teacher experience or student demographics, and there may be potential confounding factors such as curriculum changes, extracurricular tutoring. The regression model includes teacher experience and demographic agents, allowing us to estimate the impact of AI while keeping these factors unchanged. The non-significant coefficients of teacher experience (β = −0.11, p = 0.594) and demographics (β = −0.19, p = 0.433) suggest that the benefits of AI are powerful and not severely driven by these confounders.

**Table 12:** Comparison of teaching effect improvement on the east and west sides of the Hu Huanyong Line.

	Natural Sciences (Mathematics)		Social Sciences (History)	
	Improved score	Improved proportion	Improved score	Improved proportion
Improved score in west region school	19.2	28%	8.9	12%
Improved score east region school	7.1	8%	4.5	5%

## 5. Discussion

### 5.1. Analysis of experimental results

The study results clearly and unequivocally demonstrate that teaching methods assisted by AI smart devices are significantly superior to traditional teaching methods in multiple aspects. This finding undoubtedly brings new opportunities and hope for the transformation of the education sector. The more pronounced improvement in teaching quality in economically underdeveloped regions, along with higher educational investment efficiency, not only profoundly reflects the rapid progress of educational technology in the tide of the times but also highly aligns with the concept of sustainable development, painting a hopeful blueprint for the future of education.

Firstly, the introduction of AI technology acts as a leveling mechanism, delivering high-quality educational resources that transcend geographical and economic barriers [55]. In both natural and social sciences, AI-powered tools like interactive simulations and data analysis platforms lead to higher average student scores [49]. For under-resourced areas, this provides unprecedented access to learning opportunities, directly addressing long-standing disparities in educational quality and helping to narrow the urban-rural achievement gap [50,51].

Secondly, AI can help achieve personalized and data-driven teaching. AI tools function as adaptive learning systems, tailoring educational content and pacing to individual student needs [52]. This data-driven approach is particularly effective in environments with scarce teaching resources, as it allows students to engage in self-directed learning while receiving targeted support. This effectively compensates for the limitations of a one-size-fits-all traditional classroom [48,53].

Finally, the study highlights a fundamental shift in pedagogy. AI technology redefines the teacher’s role, moving them from being the primary source of knowledge to becoming facilitators of learning [56]. By providing rich learning analytics and automating routine tasks, AI tools empower educators to use real-time data to identify student challenges and offer targeted support. This enables a more personalized teaching practice and promotes the teacher’s own continuous professional development [57].

### 5.2. Theoretical contributions of the study

Research confirms that AI-powered educational tools can significantly advance educational equity, a core principle of sustainable development. By democratizing access to high-quality learning resources, AI technology offers an innovative pathway to address the goals of educational equity theories, which seek to eliminate barriers to opportunity caused by geographic or economic factors. The potential for AI to level the playing field is particularly high in underserved rural and economically underdeveloped regions.

However, realizing the potential of artificial intelligence in education requires overcoming significant implementation challenges, often referred to as the “second-level digital divide”—a gap that lies not just in access to technology, but in the ability to use it effectively [58]. This study identified several key hurdles: first is the utilization gap, where, for example, the utilization rate of VR laboratories in developed eastern regions reaches 78% while in western counties it is less than 35% due to infrastructure issues like network latency. Second is the issue of content adaptation, where the effectiveness of AI in culturally complex social science courses lags behind that of natural sciences by approximately 23 percentage points, highlighting the urgent need for content localization. Therefore, bridging the “last mile” to true educational equity requires combining technological deployment with systemic institutional innovation.

Future research can further strengthen cooperation with local governments and education departments to broaden data collection channels and extend the duration of experiments to ensure the quality and timeliness of data. In terms of research methods, although a variety of methods were used, the integration of quantitative and qualitative analyses was not tight enough, and some analyses failed to fully explore the underlying reasons behind the data. Subsequent research can further optimize research methods, enhance the coordinated use of different methods, and improve the depth and breadth of the research.

Looking ahead, with the continuous development of China’s economy and society, the issue of educational resource investment will continue to attract attention. Future research can start from a dynamic perspective to deeply study the long-term trends in educational resource investment and the dynamic adjustment mechanisms of educational resource investment in different regions. In combination with national strategies such as rural revitalization and coordinated regional development, research can explore how to optimize educational resource investment to promote educational development in rural and economically underdeveloped areas, providing strong support for rural revitalization and coordinated regional development. Strengthening the study of the benefits of educational resource investment and exploring how to improve the efficiency of educational resource utilization to achieve the maximization of educational resources will better promote educational equity and sustainable development.

### 5.3. Limitations, confounding factors, and future directions

This study highlights the potential of AI-enabled teaching devices to advance educational equity across the Hu Huanyong Line, yet several limitations and confounding factors require careful consideration. Curriculum differences between standardized mathematics and regionally tailored history content likely amplified score improvements in natural sciences. For example, in schools in western cities, the math curriculum has increased by 19.2 points, compared to 8.9 points in history. Extracurricular tutoring, more common in wealthier Eastern regions, may elevate baseline scores there, such as 85.95 in math versus 69.35 in the West. Unmeasured baseline student abilities, shaped by prior educational access, could further skew AI’s apparent impact. Infrastructure challenges, including unreliable internet in 63% of Yushu’s Western schools, likely diminished AI’s effectiveness despite randomization efforts [40]. Multivariate regression in Table 13 reveals that these confounders narrow the regional score gap by approximately 20%, with a significant regional effect, urging cautious interpretation of AI’s causal role. Incomplete student records in Western cities like Hami and Wuwei also limited data representativeness and accuracy.

To address these challenges, future studies should leverage individual-level data from the full sample of 620 students and apply propensity score matching to isolate AI’s effects from confounders like tutoring or curriculum variations [59]. Longitudinal research spanning multiple semesters, aligned with China’s rural revitalization strategy, could optimize AI deployment in Western schools. Developing devices with multi-ethnic language support and culturally relevant content, such as minority languages in Qinghai, would enhance regional compatibility [60]. Robust teacher training programs, especially in the West where equipment idling rates reach 60% due to low technical literacy, are essential for sustainable adoption [61]. These advancements, rooted in SDG4’s vision for inclusive education, will further reduce disparities and foster sustainable educational development [62].

## 6. Conclusion

This study aimed to investigate the attitudes of teachers and students in economically developed and underdeveloped regions towards the use of AI smart devices in classroom teaching and behavior analysis, as well as to analyze and present data on teaching effectiveness. Through an online questionnaire survey, we collected 247 valid questionnaires from teachers and 620 student exam scores, and conducted regression analysis to identify potential differences in attitudes and intentions among different teacher groups, as well as to compare the effectiveness of using AI smart teaching devices across eight cities.

The study revealed that the use of AI smart devices in teaching processes in economically underdeveloped regions was significantly more effective than in developed regions, with an overall improvement of 7% to 20%.

Moreover, while teachers in economically underdeveloped regions were generally positive about the introduction of smart devices, they still faced numerous challenges in operation, including insufficient familiarity with the technology, lack of teaching resources and support mechanisms, and significant technical difficulties in integrating the devices into teaching practices. This indicates that hardware donations alone are insufficient to drive sustainable educational development. To genuinely and sustainably improve teaching quality and student learning outcomes, comprehensive support measures must be implemented, especially in teacher capacity building, technical assistance, and the development of pedagogically appropriate curricula. Such support is crucial for achieving sustainable educational development and ensuring that the introduction of technology aligns with long-term educational goals.

## Supporting information

S1 Appendix Appendix A. (DOCX)
