# Supplementary material for: Research on cross-regional adaptation strategies for AI-enabled teaching devices from an educational equity perspective
Source: PLoS One. 2026 Jan 14;21(1):e0327696. doi: 10.1371/journal.pone.0327696 (PMC12803460; doi:10.1371/journal.pone.0327696)
Supplement: S1 Appendix — (DOCX) [file pone.0327696.s001.docx]

**Appendix A**

| **Item** | **Source** |
| --- | --- |
| **PEOU** |  |
| Using AI smart devices in the classroom is effortless. |  |
| I will be able to use AI smart devices in the classroom. |  |
| AI smart devices in the classroom are very convenient to use. |  |
| **PU** |  |
| Using AI smart devices in the classroom can help improve efficiency. |  |
| Using AI smart devices in the classroom can enhance my teaching effectiveness. |  |
| Overall, I believe that using AI smart devices in the classroom is very useful. |  |
| **Compatibility** |  |
| Using AI smart devices in the classroom will be compatible with my daily teaching. |  |
| AI smart device technology will fit my teaching methods. |  |
| Using AI smart devices in the classroom will suit my teaching style. |  |
| **Performance Expectancy** |  |
| I believe AI devices are very useful in teaching. |  |
| I believe AI devices can increase the efficiency of teaching work. |  |
| I believe AI devices can help analyze student classroom behavior more accurately. |  |
| **Facilitating Conditions** |  |
| I have the knowledge required to use AI teaching devices. |  |
| If I encounter any problems, I expect to call the technical support team. |  |
| I believe I can get guidance when using AI devices for teaching. |  |
| **Attitude** |  |
| I find using AI smart devices interesting. |  |
| I look forward to the application of AI smart devices in teaching. |  |
| Overall, my attitude towards using AI smart devices in the classroom is that they are very useful. |  |
| **Intention** |  |
| I hope to use AI smart devices in the classroom in the near future. |  |
| I am determined to continue using AI smart devices in the classroom. |  |
| I plan to start using AI smart devices in the classroom as soon as possible. |  |
